# Integrating Modern Technologies into Traditional Anterior Cruciate Ligament Tissue Engineering

**DOI:** 10.3390/bioengineering12010039

**Published:** 2025-01-07

**Authors:** Aris Sopilidis, Vasileios Stamatopoulos, Vasileios Giannatos, Georgios Taraviras, Andreas Panagopoulos, Stavros Taraviras

**Affiliations:** 1 Department of Physiology, School of Medicine, University of Patras, Asklepiou Street 1, Rio, 26504 Patras, Greece; sopilidis.a@gmail.com (A.S.); vkstam@gmail.com (V.S.); giorgostar14@gmail.com (G.T.); 2 Department of Orthopedics and Traumatology, Sports Medicine Department, University Hospital of Patras, Asklepiou Street 1, Rio, 26504 Patras, Greece; vasileiosgiannatos@outlook.com (V.G.); andpan21@gmail.com (A.P.)

**Keywords:** anterior cruciate ligament, anterior cruciate ligament reconstruction, scaffold, tissue engineering, artificial intelligence, stem cells

## Abstract

The anterior cruciate ligament (ACL) is one of the most injured ligaments, with approximately 100,000 ACL reconstructions taking place annually in the United States. In order to successfully manage ACL rupture, it is of the utmost importance to understand the anatomy, unique physiology, and biomechanics of the ACL, as well as the injury mechanisms and healing capacity. Currently, the “gold standard” for the treatment of ACL ruptures is surgical reconstruction, particularly for young patients or athletes expecting to return to pivoting sports. Although ACL reconstruction boasts a high success rate, patients may face different, serious post-operative complications, depending on the type of graft and technique used in each one of them. Tissue engineering is a multidisciplinary field that could contribute to the formation of a tissue-engineered ACL graft manufactured by a combination of the appropriate stem-cell type, a suitable scaffold, and specific growth factors, combined with mechanical stimuli. In this review, we discuss the aspects that constitute the creation of a successful tissue-engineered graft while also underlining the current drawbacks that arise for each issue. Finally, we highlight the benefits of incorporating new technologies like artificial intelligence and machine learning that could revolutionize tissue engineering.

## 1. Introduction

ACL ruptures constitute 45% of knee injuries, with approximately 100,000 ACL reconstructions taking place annually in the United States. Such injuries are often sustained by athletes performing high-intensity sports [1,2], with the leading treatment being surgical reconstruction with different types of grafts. Despite the high rate of success, serious post-operative complications cannot be avoided with the current form of ACL reconstruction. Surgeon experience, autograft availability, patient occupation and activities, comorbidities, psychological conditions, prior knee surgery, and the extent of the current injury are all factors majorly affecting the outcome after ACL reconstruction [3]. Factors related to the surgical technique, including graft type selection, femoral tunnel placement, and the portal used, as well as the type of femoral fixation, also affect the outcome and revision rates after ACL reconstruction [3,4,5,6,7]. Therefore, the inability of the current techniques to overcome those issues leads to the need for their optimization or the incorporation of new technologies, such as tissue engineering [8,9]

This review assesses the current trends for ACL reconstruction and cites the tissue engineering-based techniques developed for ACL tear treatment, as well as the new technologies that could be utilized in the future to relieve patients who are confronted with the problems caused by conventional ACL reconstruction.

## 2. The Traditional ACL Field as We Know It

### 2.1. Anatomy, Biomechanics, and Physiology of the ACL

**Figure 1:**
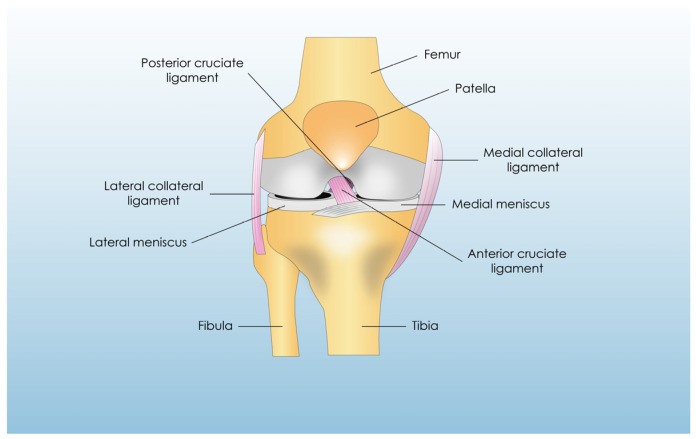
Knee joint anatomy.

An understanding of the anatomy and physiology of the ACL is crucial to construct a synthetic ACL graft using tissue engineering. The ACL is an intra-articular ligament attached to the posteromedial aspect of the lateral femoral condyle, and it descends anterolaterally to attach to the anterior intercondylar area of the tibia. The shape of the ACL itself has been a hot research topic recently, being ribbon-like, as described by the ACL Study Group in 2012 and Siebold et al. [10]. Typically, it consists of two bundles, the anteromedial bundle (AM) and the posterolateral bundle (PL), although the existence of two separate bundles or one ribbon-like that functions as two when twisted remains controversial [10]. The ACL length and width vary from 31 to 38 mm and from 10 to 12 mm, respectively [11,12,13,14,15]. In its femoral insertion, the lateral intercondylar ridge defines its anterior border, while the lateral bifurcate ridge separates the AM from the PL bundle [16,17]. These two ridges can be used as intraoperative guides for the tunnel position; however, in chronic patients, they can be absent [17]. During reconstruction, a femoral tunnel slightly anterior to the anatomical footprint is preferred in the majority of the literature as the most isometric position, leading to lower failure rates; certain authors, however, prefer a central femoral tunnel, as it leads to better graft biomechanics [18]. The tibial insertion of the two bundles, on the other hand, presents great variability among patients, with a strict or oblique anteroposterior course on the medial intercondylar ridge bisected by the central intercondylar ridge, which separates the ACL from the anterior horn of the lateral meniscus [19,20]. Tenso et al. proposed the medial intercondylar ridge and the anterior ridge as bony landmarks for the tibial tunnel placement intraoperatively [21]. The anatomical illustration of the ACL ligament and its surrounding structures is presented in Figure 1.

In order to perform a successful ACL reconstruction, it is beneficial to comprehend the biomechanics of the ACL inside the knee joint. The ACL is the primary stabilizer for the anterior transaction of the tibia relative to the femur, as it absorbs 75% of the load in full extension and 82–89% in 30 degrees of flexion. When the flexion of the knee increases, the ACL becomes horizontal to the knee, resulting in a slight decrease of the anterior load it absorbs; the AM bundle tightens, and the PL bundle relaxes [22]. On the other hand, the PL bundle is tight during knee extension, whereas the AM bundle is moderately relaxed [22]. In addition to the anterior translation, the ACL stabilizes the knee against the internal rotation of the tibia while subjected to multidirectional forces [13,14,23]. Those functions can occur due to the ACL’s specific properties, the main ones being its unique tensile strength/load, linear stiffness, and viscoelastic nature (under low strain, it demonstrates elastic behavior; however, under high strain, it has a viscous behavior). Specifically, Woo et al. measured the maximum tensile load and linear stiffness. The results varied depending on the age group. The values for younger specimens (under 35 years) were, respectively, 2160 ± 157 N and 242 ± 28 N/mm, and they drop dramatically with age [24,25].

The ACL as a structure is similar to other types of connective tissue. Its dry weight consists primarily of Extra Cellular Matrix (ECM) plus specific cells. Microscopically, it is important to comprehend that the cellular physiology and ECM consistency differ depending on the specific zone (proximal, middle, or distal) of the ACL. The ECM is a non-cellular component comprising (a) a collagen matrix organized in fiber bundles (90% type I, 10% type III, and other minute types), (b) glycosaminoglycans (GAGs)-proteoglycans (their absorption of water contributes to the viscoelastic properties of the ACL), (c) other extracellular proteins (such as fibronectin, tenascin, and laminin), and (d) elastin. The cell components are (a) mostly fibroblasts of various shapes and sizes and (b) chondrocytes [12,13,26].

### 2.2. Injuries and Healing of ACL

The ACL is one of the most commonly injured ligaments in both male and female patients. The mechanism of the injury can be due to contact or non-contact (force generated by the patient’s movements). Contact injury can be further divided into direct contact to the knee and indirect contact, meaning an action on a body part that affects the knee. As far as non-contact ACL injuries go, which account for the majority, they are caused by sudden forward movement of the tibia relative to the femur (e.g., sudden deceleration movements) and twisting of the tibia relative to the femur (e.g., pivoting/change of direction movements) [27,28,29,30].

The healing of the ACL is considered to be poor. The healing process is almost identical to that of other connective tissue structures (inflammation, epiligamentous regeneration, proliferation, and remodeling), with the exception of one phase, the epiligamentous reparative phase. According to M.M. Murray et al., this phase is probably responsible for the poor repair due to the formation of a synovial tissue layer that covers the ends of the ruptured ligament [31]. When assessing the healing potential except for the histological changes, other environmental and innate factors need to be taken into consideration. Some of them are (a) the movement of the synovial fluid surrounding the ACL inside the knee joint, which inhibits the formation of a blood clot, resulting in the absence of a provisional scaffold that does not bridge the two ends of the ruptured ligaments; (b) the decreased amount of ECM protein in the rupture site; and (c) the low vascularization of the ACL and its dull response to the wound in contrast to the vascularization of other ligaments [31,32,33,34,35].

### 2.3. Current Techniques Utilized for Treating the Ruptured ACL

When dealing with a ruptured ACL, an appropriate approach should be followed. The physician’s options are either conservative or surgical treatment, with the “gold standard” method being the latter. As far as the conservative strategy is concerned, it can yield benefits to specific groups of patients through neuromuscular rehabilitation. Those patients are either copers (those who can be functional with an ACL deficiency) or adapters (those who can alter their activities to evade surgery). Also, this approach offers positive results to patients who decide to engage in non-competitive or non-contact sports and low-demand daily activities. However, young patients at risk for developing osteoarthritis or patients participating in pivoting sports are strongly recommended to have ACL reconstruction. Despite all of the above, clinical evidence is still lacking for comprehensive clinical guidelines [27,28,36,37,38,39].

In the field of orthopedics, ACL reconstruction is an extensively analyzed operation, with articles being constantly published about the advancements in each different aspect of the procedure. A bibliometric review in 2022 found 12,223 articles published on ACL reconstruction, with a rapidly increasing research trend since 1990 [40]. ACL reconstruction provides satisfactory results (ranging from 75 to 97%) as far as stabilization of the knee joint, mitigation of symptoms, and return to normal daily activities are concerned [41,42,43]. Several factors, however, as mentioned above, influence the outcome and revision rates.

A typical ACL reconstruction consists of the following stages: (a) preparation of the patient using anesthesia, (b) selection and harvesting of the graft material, (c) graft preparation that includes suturing the autograft and applying pretension to it, (d) formation of the bone tunnels/sockets inside the femur and tibia, (e) placement of the graft inside the tunnels/sockets, and (f) graft fixation. Each of the above stages has been meticulously studied in order to piece together the best possible result for each patient [43,44,45]. Additional procedures like lateral extra-articular tenodesis are utilized by many surgeons, with a lack of official guidelines for the time being and the choice being a matter of personal preference [46]. Despite those efforts, some problems arise, given the fact that several studies have proven that revision surgery due to complications or graft failure occurs. ACL reconstruction failure in need of revision surgery is defined by Noyes and Barber-Westin as (a) complete graft tear with anterior drawer test more than 6 mm compared to the healthy side and (b) positive pivot shift test grade II or III in the presence or not of knee pain, functional limitation, and sense of instability [7]. The percentage of patients in need of ACL revision surgery fluctuates between 2.9 and 5.9%, regardless of their athletic background. However, other studies suggest that this percentage is even higher (10–15%) [41,42,47]. The symptoms and complications that correspond with ACL reconstruction failure, leading to a potential ACL revision surgery, are divided into instability, stiffness, and pain in the patient’s knee. As for the underlying reasons for ACL reconstruction failure (rupture or functional failure of the graft) and its post-operative complications, they can be grouped into the following categories: (a) flawed surgical technique (77–95%), with the most common error being the non-anatomic tunnel drilling; (b) acute or chronic trauma (5–10%); and (c) biological factors (e.g., graft biocompatibility problems or infection). The above categories can be further specified, as each one of them includes a plethora of conditions. The most important conditions, which are the subject of study in reference to ACL reconstruction’s poor outcomes, are false graft choice and osteoarthritis [26,41,42,48,49].

The selection of the most suitable graft for each distinct case of ACL reconstruction has been an ongoing debate in the scientific community. The main type of grafts used in ACL reconstruction nowadays are autografts. However, other types of grafts have also been used, such as synthetic grafts and allografts. Synthetic grafts are scarcely used due to severe complications, including biocompatibility problems (foreign body inflammation), material degradation, and wear debris, leading to poor clinical outcomes (high graft rupture percentage) [41,42,45,50]. Allografts also have a higher failure rate than autografts and worse clinical outcomes (instability of the knee) due to slow revascularization of the graft, leading to poor graft incorporation and the minute risk of disease transmission (1/1,000,000). Further sterilization utilizing radiation could be detrimental, diminishing the graft’s mechanical properties. The main reason for their utilization is the absence of donor-side morbidity, rendering them a good choice for multiple ligament injury and revision surgery [41,42,43,51]. As far as autografts are concerned, there are the following options: hamstring tendon, patellar tendon, and quadriceps tendon. Many studies have been conducted comparing the three different tendon grafts’ extraction techniques and post-operative complications. The patellar tendon and the hamstring tendon are the most commonly used grafts for ACL reconstruction, with either one of them achieving greater biomechanical characteristics than the native ACL. Nevertheless, the hamstring tendon grafts indicate higher rupture rates than the patellar tendon grafts, with a probable cause being graft slippage. The bony attachments of the patellar tendon grafts promote further incorporation of the graft, which could be associated with less rehabilitation time. However, removing part of the patella could lead to donor-side morbidity, patellar fractures, decreased extension strength, and anterior knee/kneeling pain. On the other hand, utilization of HT grafts exhibits less donor-site morbidity (significantly lower anterior knee pain) and a better cosmetic outcome, but they are associated with saphenous neuromas, decreased flexion strength, and potential graft rupture during harvesting [15,42,43,51,52,53,54,55]. An up-and-coming alternative graft has been the quadriceps tendon. A meta-analysis from Mouarbes et al. has shown that a quadriceps tendon autograft displays comparable clinical outcomes to the patellar and hamstring tendon, providing a suitable graft with sufficient graft size, significantly less knee pain during the short-term follow-up than in a patellar tendon ACL reconstruction, and better results in clinical trials than a hamstring tendon ACL reconstruction [56]. All in all, autografts are the mainstay of graft choice during ACL reconstruction, but the harvest site remains a personalized decision per patient needs, as they show similar clinical performance [57]. Sprinters would avoid a hamstring graft, and kneelers would avoid a patellar tendon graft, but the expertise of the surgeon on the particular grafts should also play a role in the decision-making [57]. The grafts used in ACL reconstruction currently, along with the complications they are accompanied by in the literature, are summarized in Table 1.

The purpose of ACL reconstruction is the restoration of knee motion and the obstruction of post-traumatic osteoarthritis development, which is established in 50–90% of patients [27]. No matter the technique chosen for ACL reconstruction, post-traumatic osteoarthritis is still a major concern in a respectable percentage of patients, with an increasing risk over time [58]. A recent thorough study by Bodkin et al. showed that roughly 12% of patients develop post-traumatic osteoarthritis five years post-operatively [59]. Strong evidence, however, is still lacking regarding the prevention of OA by ACLR [27].

## 3. Tissue Engineering: Panacea for ACL’s Reconstruction Issues?

**Figure 2:**
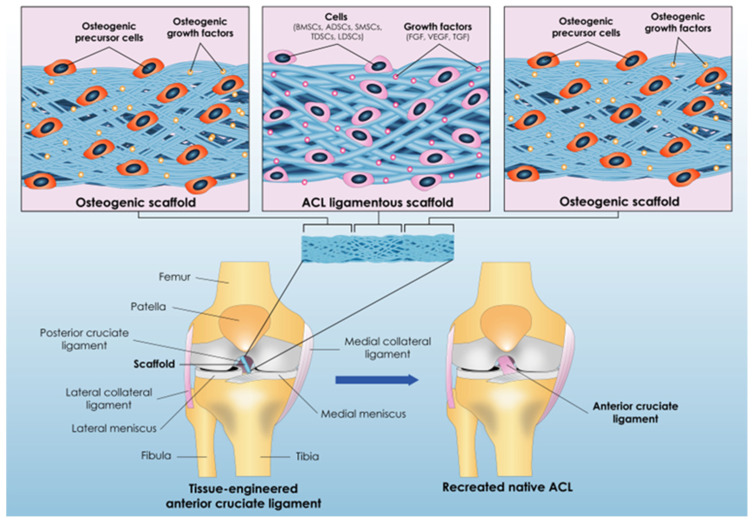
ACL tissue-engineering approach. The left image is a ”zoomed in” representation of the micro-environment of the scaffold–stem cells–growth factors combination that is implanted in the knee joint, with the right image being the end result of the tissue-engineering approach, which is a new recreated ACL similar to the patient’s ACL before the rupture. The scaffold portrayed has three parts/phases. The two sides of the scaffold constitute its anchors that will be attached to the femur and the tibial bone, respectively, and the center of the scaffold is the one that will create the new ACL ligament.

From the above mentioned, the path of developing the most suitable/less problematic approach for combating ACL ruptures led to the initiative of creating an engineered ACL graft utilizing stem cells that mimics the native one’s properties. This promising approach, called ACL tissue engineering, could upgrade the field of ACL reconstruction and allow it to deviate from the standard reconstruction techniques. Tissue engineering is a multidisciplinary field that incorporates harmonically the combination of (a) cells, (b) scaffolds, and (c) biologically active molecules with the aim of formulating a functional tissue that simulates the physiology, function, and morphology of the native one. The depiction of the ACL tissue-engineering approach is demonstrated in Figure 2.

### 3.1. Issue No1: The Appropriate Scaffold Construction

Since the stem cells constitute the building blocks of the tissue-engineering technique, scaffolds could be characterized as “the foundation” of the tissue-engineering approach. Their role is dual, meaning that a scaffold should be manufactured in regard to both the macroscopic and microscopic aspects of the native ACL tissue. As mentioned before, the ACL has a very intricate structure both “externally” and “internally”. Those two facets of the scaffold architecture refer to (a) the specific length, width, and function of the ACL (in regard to the external) and (b) the complex alignment and composition of the ECM fibers which promote tissue regeneration (in regard to the internal) [60,61].

The typical approach, used in the majority of experiments, has two main steps, the first one being the method of choice for the creation of the scaffold structure and the second one being the selection of the desired biomaterial(s) [62,63,64,65]. The wide variety of compilations of the two above elements is evaluated based on the scaffold’s final morphology, its achievement of the mechanical properties of the native ACL tissue, and its biodegradability and biocompatibility [60,66].

#### 3.1.1. The Process of Creating the Scaffold’s Structure

The morphological basis of scaffolds is fibers of different biomaterials. The fibers of fibrous scaffolds can have aligned or random orientation [67]. The most widely used method for fiber construction is electrospinning [66,68,69], while new technologies like 3D bioprinting are starting to emerge [70]. Electrospinning is an inexpensive, reliable method of fabricating fibers similar to natural ones with a variety of tailorable parameters providing a sufficient surface area for cell attachment and also high porosity. However, it may be toxic for cells due to organic solvents used in the process, such as dimethylformamide and chloroform [67,71]. Electrospun fibers can be manufactured in many forms, such as knitted and braided scaffolds [62,72]. For instance, the properties of a braided scaffold can be influenced by the braiding parameters such as the angle, the pattern, and the height of the braiding [62,72]. Three-dimensional bioprinting, on the other hand, is a low-cost, high-efficiency fabricating method that creates primarily high-resolution 3D structures combining cells and different materials, such as hydrogels, which constitute a more suitable environment for cell proliferation. Nevertheless, the absence of a variety of bio-inks limits its use [73].

The critical parameters in tissue engineering, when developing the morphology, are the appropriate pore size and porosity that are aimed at tissue regeneration and simultaneously successful cell attachment. In particular, for soft tissue, it has been found that a 200–250 μm pore size is acceptable for tissue ingrowth [74]. The development of pores targets the creation of a suitable microenvironment and should match certain criteria: (a) the pores should be interconnected to promote cell proliferation and free diffusion of fluid, and (b) the pores should have an optimal size and number for allowing directed cell proliferation while still providing sufficient surface area for cell adhesion and maintaining mechanical support [75,76,77].

When finally creating the desirable morphology, it is of the utmost importance that the created structure achieves the replication of the initial ACL properties mentioned in the biomechanics section (tensile strength, linear stiffness, and viscoelasticity). Those measurements are typically estimated in vitro [3,24,78]. However, it is important to calculate the forces that the ACL is subjected to in common, everyday activities that are straining the ACL from multiple directions. In a study by Roldan et al., the maximum tensile force acted on the ACL during everyday activities was calculated to be 513.068 ± 8.337 N in walking, 726.003 ± 64.222 N in jumping with both legs, and 368.243 ± 113.627 N in running [79].

Biodegradability is a factor that drastically affects the scaffold’s mechanical properties after in vivo implantation. The ACL is an intra-articular ligament that is surrounded by synovial fluid, which consists of many proteolytic enzymes that degrade materials placed in its environment, and it is also submitted to constant mechanical stimuli. Thus, the scaffold implemented should be able to withstand these conditions until adequate cell proliferation and ECM production occur by the stem cells. During the degradation, its mechanical strength deteriorates; hence, this reduction should be compensated by the newly produced ECM. Theoretically, this equilibrium between scaffold resorption and new tissue growth should be “perfectly balanced” in order to achieve the required mechanical properties throughout the whole process of new ACL development [61,70]. It is obvious that biodegradability is a major aspect affecting the success of an ACL tissue-engineered graft. This is the reason researchers have solely tried to predict/understand the parameters and details that affect the resorption time of each type of scaffold. Studies conducted examining biodegradability are performed either in real-time conditions [70] or, recently, utilizing computational tools in order to predict the outcome without conducting the full-fledged experiment process [80,81].

#### 3.1.2. Constructing the Scaffold’s “Anchors”

At present, ACL reconstruction surgery utilizes classic orthopedic techniques for the graft’s fixation, thus canceling the ligament-to-bone interface observed in the native ACL. Tissue engineering could offer a possible solution, trying to recreate the natural ligament-to-bone interface. This interface, called “enthesis”, is the region where the ACL attaches to the bone, and it contributes to the smooth force transition between the ACL and the bone. Enthesis comprises four continuous but distinct areas with different characteristics, them being the ligament zone, the non-mineralized fibrocartilage zone, the mineralized fibrocartilage zone, and the bone zone. Each zone differs from the other in terms of cell types, ECM composition, and structure, thus resulting in different mechanical/biological properties. Specifically, (a) the ligament zone has the physiology and composition of the ligament discussed above, (b) the non-mineralized fibrocartilage zone consists of chondrofibroblasts, (c) the mineralized fibrocartilage zone consists of hypertrophic chondrocytes, and (d) the bone zone consists of osteoblasts, osteoclasts, and osteocytes. Due to the complexity of creating a ligament-interface-bone scaffold, the challenge remains to encompass all the distinct areas while allowing each one to have its unique nature/function. This should also be achieved with the perspective of allowing each area to interact with each other similarly to the enthesis of the native ACL [82,83,84,85]. When trying to achieve this task, each “phase” of the scaffold should be manufactured according to the zone of the scaffold that we want to recreate. This means that each area should have different parameters followed (different materials, manufacturing techniques, and cells) in order to create this zonal differentiation [86]. For this overlapping environment to occur, multi-phasic scaffolds must be created that abide by the characteristics discussed above (fiber orientation, mechanical properties, pore size, and biodegradability rate) for each phase. This task is extremely complex in the field of ACL tissue engineering on its own, and it still poses a challenge for the future.

#### 3.1.3. Biomaterial Selection

Following the comprehension of the parameters for the creation of a proper ACL scaffold, it is still required to resolve which should be the ideal biomaterial that can be formed in the desirable shape while having proper mechanical properties, biodegradability rate, and biocompatibility. The materials utilized are natural and synthetic polymers, which can also be combined into a composite scaffold.

i. Natural biomaterials

Ninety percent of the ACL’s dry weight consists of type I collagen. That fact led to the use of collagen as the primary candidate for ACL scaffold fabrication. Collagen is a biocompatible material with multiple adherence sites for fibroblasts to proliferate [87]. Excluding its innate biocompatibility, it demonstrates a high degradation rate and poor mechanical strength [66,88]. Those issues were addressed due to the easily modified nature of collagen by the crosslinking of collagen fibers using chemical agents or physical methods such as UV light. However, the chemical agents used in crosslinking could be potentially toxic to the joint. Other possible drawbacks of collagen are its high cost and possible disease transmission [89,90,91]. Comparable to collagen, silk is another natural material with a variety of applications in medicine (such as suture material) that has been utilized previously in ACL tissue engineering [92,93]. Silk fibroin is also a biocompatible choice, provided that the layer of sericin (a glue-like protein accounting for hypersensitivity problems) is extracted, which promotes bone marrow mesenchymal stem cell adhesion, proliferation and differentiation (expression of ligament markers) [94]. The main reason why silk is considered a viable candidate for ACL tissue engineering is its comparable tensile properties to the native ACL while being modifiable. Specifically, it can be shaped into foams, gels, and fibers. The alignment of the fibers influences the scaffold’s mechanical properties even more. Another advantage of silk is genetic modification, which alters its composition, defining its properties [66,89,91]. Moreover, unlike collagen, silk exhibits a low degradation rate that varies according to the scaffold’s attachment region and the patient’s general condition. However, the general consensus is that silk loses its tensile strength in vivo in one year and completely degrades in two years. Silk can cause problems when sericin is not properly removed and also elicits the danger of the formation of granulomas [94]. Except for those two leading natural biomaterials for ACL tissue engineering, other choices have been proposed, such as chitosan, alginate, and hyaluronic acid. However, they are not considered viable options for scaffold fabrication on their own due to the major issue of inadequate mechanical strength [66].

ii. Synthetic biomaterials

An alternative way is the use of synthetic, biodegradable biomaterials, which are highly tailorable (the above parameters of the scaffold can be determined via different polymer solution variations) and can be used in ligament tissue engineering. Poly(lactide-co-glycolide) (PLGA) is a commonly used biomaterial that comprises lactic acid (LA) and glycolic acid (GA). The LA/GA ratio can be altered, thus modifying the PLGA’s final properties, rendering it a highly tailorable option. Specifically, its degradability varies depending on the above ratio. A 50:50 PLGA degrades in 1–2 months, a 75:25 PLGA degrades in 4–5 months, and an 85:15 PLGA degrades in 5–6 months [95]. Moreover, bone marrow mesenchymal stem cells attach and proliferate at high rates on PLGA, especially in the high molecular weight one (50:50) [96]. The major drawback is its acidic degradation, which causes inflammation in the installation site [91]. Comparable to PLGA, poly L-lactic acid (PLLA) is a biomaterial used widely in orthopedics due to its superior mechanical strength, and it exhibits higher cell adhesion capability than PLGA. Despite its slow degradation rate, PLLA has a unique feature: it loses its mechanical strength in a 6-month period when hydrolyzed, while no significant change in its mass occurs until years after implantation. Acidic degradation is still, in this case, a significant issue [95,97]. Polyglycolic acid (PGA) is a simpler molecule biomaterial that is mainly suggested because of its high initial tensile strength. Nevertheless, it degrades rapidly in vitro, resulting in a less durable scaffold [95,97]. Last but not least, polycaprolactone (PCL) is also being widely investigated for tissue-engineering applications, mainly as part of a composite scaffold. Its major attribute is its high elongation range (20–1000%), which could be useful in a mix of materials to mimic the viscoelastic nature of the ACL. Furthermore, it degrades slowly in vivo and presents a modest immune response due to its acidic degradation [68,98].

iii. Composite biomaterials

From the above mentioned, it is apparent that neither natural nor synthetic biomaterials meet the requirements necessary for an ACL scaffold on their own. Due to this realization, research has been focused on the fabrication of scaffolds from different combinations of materials. Composite scaffolds are made with the principle of exploiting each biomaterial’s useful aspects and combining them together in an effort to create a structure that can withstand the knee’s environment and promote cell growth and differentiation. The possible combinations are natural-based composite scaffolds, synthetic-based composite scaffolds, and natural–synthetic composite scaffolds. Bi et al. developed a composite silk–collagen scaffold (group 1) and compared it to a semitendinosus autograft (group 2) after ACL reconstruction in two groups of New Zealand rabbits. The created scaffold had an outer layer of knitted silk fibroin mesh (used for its mechanical strength) and an internal collagen sheet core (used for its capability for cell ingrowth). The tests were performed after 4 and 16 weeks post-operatively in both groups. The results indicated that the failure load in the scaffold group was superior after four weeks with no major difference in stiffness, albeit at the 16-week mark, the failure load presented no significant difference, and stiffness was greater in the scaffold group. Moreover, it was proven that this type of scaffold was infiltrated by fibroblast-like cells, thus promoting tissue ingrowth [92]. Another similar study by Bi et al. proved that the incorporation of bone marrow mesenchymal stem cells into the silk–collagen scaffold further improves its potential for ligament tissue ingrowth compared to the only scaffold approach [93]. S. Sahoo et al. developed a knitted, degummed silk scaffold coated with fine electrospun PLGA fibers infused with basic fibroblast growth factor. The silk core was selected due to its superior mechanical properties, while PLGA ameliorated bone marrow mesenchymal stem cells’ adhesion and proliferation. The in vitro results backed up their assumptions that this material combination scaffold could be used further in ligament tissue engineering [99]. Ya Tang et al. constructed a biphasic composite scaffold from different ratios of PLGA/PCL (1:1, 1:2, and 1:5). The results led to the conclusion that the addition of PLGA increased the biodegradability, while the addition of PCL increased the mechanical strength. Regarding cell viability, differentiation, and adhesion, the scaffolds seeded with MSCs and placed under tension (dynamic culture) showed better performance than those without mechanical stimulation. Overall, the scaffold with 1:5 PLGA/PCL showcased the most suitable properties, similar to native ACL [65]. In a recent study, Aminatun et al. tested a nanofibrous electrospun, composite PLA:PCL scaffold in mechanical properties (tensile strength, modulus of elasticity), degradation rate, and cytotoxicity (for cell viability). The different material ratios were (A)100:0, (B) 85:15, (C) 80:20, (D) 70:30, and (E) 0:100. The results indicated that, although the scaffolds did not match the values of the ultimate tensile strength and elastic modulus of native ACL, they could be proven useful due to their degradability and cytotoxicity. The degradability test showed that the addition of PCL slowed down the degradation rate of the scaffold; thus, all groups except group A qualified for ACL reconstruction, which lasted six months. Finally, all groups were deemed non-toxic with a cell viability of over 60% [68].

### 3.2. Issue No2: The Suitable Cell Type of ACL Tissue Engineering

There are many cell “candidates”, such as fibroblasts (from the ACL or other tissues) and mesenchymal stem cells, with the most widely studied being the latter. Stem cells are the building blocks of the TE technique as they have the ability to self-proliferate and differentiate into the desired native cells, thus synthesizing the ACL ligament. More specifically, the most commonly utilized types are those derived from the bone marrow (BMSCs), the adipose tissue (ADSCs), the synovium (SMSCs), and those from tendons (TDSCs) or ligaments (LDSCs).

BMSCs are immunocompatible, inexpensive, and easily obtainable, but they are scarcely present in a bone marrow aspiration comprising only 0.01–0.001% of the cell content and carry the risk of ectopic ossification due to the existence of bone progenitors. ADSCs are also immunocompatible and can be acquired from adipose tissue with an easy and low-cost process while being in abundance (1%). However, they require processing with enzymes [50,100,101]. TDSCs are proven to demonstrate “epigenetic memory” from their originating tissue, allowing them to proliferate/differentiate easier when placed in a similar tissue [101]. Finally, LDSCs have shown the tendency to differentiate into ligament cells [102]. The last two candidates, however, have low availability in their tissues of origin.

In order for the above cells to be suitable for ACL tissue engineering, they need to meet certain standards/parameters that have been used in a wide array of comparative studies. These are (a) adequate proliferation rate, (b) adhesion and proliferation on a variety of scaffolds, (c) ECM excretion (comparative to the consistency of the native ACL e.g., GAG excretion and collagen production), (d) gene expression and protein production of ligament-related ECM markers, (e) survivability (in the native ACLs environment), (f) suitable morphology, (g) low immune response (immunocompatibility), and (h) cell fate (inside the knee joint) [103,104,105,106,107].

Ge et al. compared the potential of rabbit BMSCs, ACL fibroblasts, and medial collateral ligament fibroblasts in four major aspects: cell proliferation, density of collagen I and III, and a-smooth muscle actin expression and collagen excretion, and concluded that BMSCs are superior in each category [103]. A study by Liu et al. also proved the superiority of rabbit BMSCs against ACL fibroblasts, as their performance in each category exceeded the one from ACL fibroblasts. Those categories were cell proliferation (>7 days), GAG excretion (≥14 days), transcript levels of ECM genes of collagen type I/III (days 7–14), expression of ECM proteins of collagen type I/III (days 7–14), and histology characteristics [104]. Another study by Liu et al., comparing rabbit SMSCs to BMSCs using similar criteria as the previous one, demonstrated the superiority of SMSCs over BMSCs [108]. As for the utilization of ADSCs, Eagan et al. studied the capabilities of ADSCs regarding their ability to express multiple ligament markers under growth factor stimulation. They demonstrated that even after four weeks of transforming growth factor beta-1/Insulin-like growth factor 1 treatment, the ADSCs were unable to continuously produce collagen type I/III, leading to the assumption that their differentiation potential in ligament cells is subpar compared to other types of mesenchymal stem cells [109]. TDSC studies are scarce; however, a study by Tan et al. showed that rabbit TDSCs proliferate faster and indicate higher multilineage differentiation than rabbit BMSCs in vitro [110].

### 3.3. Issue No3: Facilitating the Right Combination of Growth Factors (GFs) and Mechanical Stimuli

Complimentary to the cells and an appropriate scaffold, there is a need to guide the differentiation of stem cells into ACL cells and enhance the production of ECM and their proliferation and neovascularization. The aforementioned could be achieved via signaling the cells with chemical molecules (GFs) or/and via mechanical stimuli [88,111]. The most prevalent GFs studied for ACL tissue engineering are fibroblast growth factor (FGF), platelet-derived growth factor (PDGF), transforming growth factor (TGF), and vascular endothelial growth factor (VEGF) [66,88]. GFs can be administered in different concentrations in cell cultures. However, due to the GFs’ limited activated time in cell media, new doses of GF need to be administered periodically. Therefore, it is imperative to discover a technique that reinforces the effect of GFs. One solution for this problem could be the modification of the scaffold’s surface, utilizing GF conjugation in order to promote cell-surface interaction. Studies have suggested that the creation of biomimetic scaffolds where GFs are conjugated chemically onto the surface of the scaffold and thus are slowly released into the pores over a prolonged period of time enhances further the formation of new ligamentous tissue [99,112].

As far as mechanical stimuli are concerned, it is already proven that the ACL is a dynamic structure in the knee joint that is constantly under strain. Previous studies have shown that the immobilization after ACL reconstruction has adverse effects on the ligament’s mechanical properties. Thus, a rehabilitation program with early mobilization of the knee joint is important for the patient’s quick and smooth recovery [51,113]. The above-mentioned is reinforced by studies proving the effect of mechanical stimuli on the cellular level. It has been found that mechanical stimulation augments cell proliferation and ECM production. Moreover, stem cells, under certain mechanical strain, present an upregulation in ligament-specific markers and tend to differentiate into ACL fibroblast cells even without the addition of GFs. Different types of mechanical strain effect uniquely different cell types. Consequently, the environment of the ACL should be replicated in order for the newly regenerated tissue to be as similar as possible to the native ACL. Bioreactors serve this purpose by simulating the knee’s unique environment while allowing the researchers to intervene with the graft’s conditions. In detail, bioreactors are used for their faculty of applying mechanical stretch with precision to the ACL graft in a controlled, sterile environment as a means to induce cell differentiation and ligament-like tissue formation [114,115,116]. Last but not least, cell stretching (primarily cyclic stretching) has been shown to provide a positive anabolic response and upscaled expression of tendon-related markers in ACL-derived fibroblasts [117]. Also, the reaction profile of the ACL fibroblasts when cyclically stretched seems promising, as it increases the collagen fiber formation/organization as well as tissue tensile properties [118]. Much research has been carried out for the discovery of the perfect combination of a mixture of GFs and a particular pattern of mechanical stimulation on a scaffold seeded with stem cells for the purpose of recreating the perfect conditions for ACL-like tissue ingrowth. However, no clear consensus has been established [119,120].

## 4. Discussion

At the present time, no clinical trials have been conducted encompassing all the aforementioned elements regarding the complete ACL tissue-engineering reconstruction that has been studied in animal models. However, there are existing clinical applications or trials that utilize each of the three aspects of tissue engineering (stem cells, scaffolds, and GFs) independently or in combination with each other. In particular, Wang et al. performed a single injection of allogenic mesenchymal progenitor cells with or without hyaluronan in 17 patients, post ACL reconstruction. This study showed that the addition of mesenchymal progenitor cells was safe and well-tolerated over a 2-year period. Also, patients with an injection of mesenchymal progenitor cells + hyaluronan exhibited better function of the knee joint as well as lower pain and higher daily activity. Furthermore, this study suggests that stem cells could ameliorate post-reconstruction complications [121]. Another clinical trial by Perrone et al. combined a collagen-based scaffold with primary suture ACL repair and concluded that although the scaffold was well tolerated by the patients, at the 3-month benchmark it lacked the mechanical strength of a hamstring autograft. This study indicates that since collagen is well-tolerated in the knee joint, it is a well-suited candidate for further clinical studies on the topic of ACL tissue engineering to be performed [122]. A meta-analysis by Zhu et al. found that platelet-rich plasma injection after ACL reconstruction improves post-operative pain and knee function early but not after a long period of time [123]. Another meta-analysis by Gong et al. demonstrated that platelet-rich plasma may improve GAG maturation but has no effect on bone tunnel widening or the improvement of knee function [124]. Overall, the injection methods cannot be directed in an intra-articular environment, rendering them unreliable; thus, they can only be used in certain groups of patients, with possibly positive results for each patient, complementary to the ACL reconstruction surgery.

It has been obvious that the application of complete tissue engineering is a complex subject that requires the calculation of a wide variety of intertwined parameters that arise when trying to achieve the recapitulation of the native ACL at the preclinical level. Thus, it is of the utmost importance that the current form of tissue engineering is reinforced by new technologies that allow the minimization of the existing problems. One method that may limit the negative results of the typical trial-and-error approach is the utilization of computational tools. Laurent et al. created a multilayer braided PLCL scaffold and inserted its architecture into an algorithm (Finite Element code) which compared the geometry of the computed version of the scaffold versus the actual one and then compared the measurements of the computed PLCL scaffold versus tensile tests from actual experiments in different scaffolds, thus predicting its mechanical properties. The Finite Element code measures the scaffold’s microenvironment by calculating each fiber’s course and its interaction of friction with other fibers. Therefore, the cell activity in the scaffold could be interpreted. Moreover, such tools could predict the best architecture for recapitulation of the native ACL while bypassing separate tests for each different architecture [125].

As time progresses, the utilization of new technological means in tissue engineering becomes more common, as it improves efficiency and further restricts the use of the empirical/trial-and-error approach. Artificial intelligence (AI) and machine learning (ML) have come to the forefront of medical research, as well as tissue engineering, with the number of studies applying those tools exponentially increasing in recent years. They provide powerful tools that use databases for the optimization and prediction of various aspects, with their impact already implemented in the orthopedics department, especially in the field of ACL injury [126,127]. Ye et al. used ML analysis to predict the clinical outcome of 432 patients after ACL reconstruction and demonstrated that ML could be beneficial in pre-operation decisions for the planning of the surgery [128]. Moreover, a review from Andriollo et al., through examination of the recent literature, showcased the clinical relevance that these new technologies can possess in all aspects of ACL injuries, specifically the prediction of ACL injury/re-injury, post-operative rehabilitation, and diagnosis of ACL tears [127].

Despite their recent utility in ACL injuries, ML and AI have yet to be used in ACL tissue engineering. This gap in the field is important to highlight, as these advanced technologies could prove to be powerful allies for the optimization and prediction of various aspects discussed above, making the current complex challenges that humans cannot overcome manageable. This particular point is validated due to the fact that the implementation of AI and ML is utilized in other tissues in the field of tissue engineering, providing promising results. Rafieyan et al. used 28 ML algorithms individually and concurrently in order to assess different scaffolds in cardiac tissue engineering by predicting the cell differentiation and behavior in each one. The researchers concluded that these ML algorithms could accurately predict the cell behavior in the scaffolds (up to 93% accuracy) [129]. Another study, by Conev et al., aimed to optimize the 3D bioprinting technique by speculating the quality of 3D bioprinted scaffolds used for bone TE. The ML software (based in Random Forest models) used polymeric biomaterials and different printing configurations as inputs to determine if the resulting product quality was high or low. The outcome of the study was that the methods utilized were able to correctly label the majority of the tested configurations [130]. Other studies have tried to predict different properties of scaffolds using ML and AI machines. Barrera et al. trained an ML machine to be able to predict the mechanical properties of a scaffold after it is inputted as images by virtual tomography into the software. Specifically, they used virtual tomography to create layered 2D images of a collection of scaffolds (dataset), and they provided each scaffold’s mechanical properties to the machine. In this way they trained it to be able to predict the mechanical properties of new scaffolds inserted into the software in the form of 2D-layered images [131]. Furthermore, Entekhabi et al. utilized an ML-based software for the prediction of the degradation rate of genepin cross-linked gelatin scaffolds. The results were promising, with a 2,68% mean squared error [132]. An overview of the AI-ML methodologies and their results discussed above is summarized in Table 2.

Taking all the above into consideration, it is clear that AI and ML could also be applied in ACL tissue engineering, providing rapid advancements in this field. Each aspect of the creation of a tissue-engineered ACL can possibly be optimized by these technologies. Specifically, discovering patterns among the literature, AI, and ML could optimize the fabrication process of a scaffold by determining the perfect parameters for its fabrication process and its architecture while predicting several other aspects that were previously discussed that create the novel scaffold. Furthermore, this approach could discover a process that has yet to be determined about the quantification/effect of different types and concentrations of GFs together with the right pattern of mechanical stimuli. Last but not least, their capability of continuous learning yields constant improvements in the long run. However, for ML software to be utilized at its full potential, there is a need for large datasets of different types of data for each aspect that is crucial for the tissue-engineering process because, up to this point for both, the amount of data is limited, not permitting a standardization of the methodology due to insufficient datasets [133].

## 5. Conclusions

Tissue engineering is a promising field with great potential in solving the problems caused by regular ACL reconstruction surgery. However, further research is needed in order for tissue-engineering techniques to be applied in the surgical treatment of ACL rupture, as it requires the mastery and complete understanding of a lot of intricate core aspects that were discussed above. The integration of new, rapidly advancing technologies like AI and ML techniques into the field of tissue engineering creates an opportunity for new accelerated, more efficient, and low-cost research on aspects that prevent the use of tissue-engineered ACL grafts for reconstruction surgery. The results of this research could possibly lead to a different perspective in tackling ACL ruptures as grafts are tailored specifically for each patient. Problems such as donor-site morbidity, long rehabilitation periods, and post-operative osteoarthritis will eventually be vanquished, leading to clinical outcomes with better results and decreased risks.

## Figures and Tables

**Table 1:** Complications reported in the literature for each graft type in ACL reconstruction, creating the need for further development of graft options.

	Hamstrings	Bone–Pattelar-Tendon–Bone	Quadriceps Tendon	Allograft	Synthetic
Complications	Higher rupture rate than patellar tendon [53], Saphenous neuromas [57], Decreased flexion strength [57], Unpredictable graft size [57], Increased tunnel widening [57], and knee laxity [53,54]	Anterior/kneeling knee pain [57], Patellar fractures [57], Decreased extension strength [54], Graft-tunnel mismatch [55]	Technically demanding [56], Post-operative hematoma [56], Donor-site pain [56], Decreased extension strength [56]	Slow revascularization [57], Poor incorporation [57], Residual instability [55], Higher failure rate than autografts [55], Risk of disease transmission [57], High cost [57], Sterilization further increases graft failure [55]	Foreign body inflammation [57], Material degradation- wear debris [57], Higher graft rupture rate than autograft [57]

**Table 2:** An overview of the discussed AI-ML technologies utilized in tissue engineering and ACL injuries with their results.

Study Group	Technology Utilized	Purpose	Function of the Program	Results
Rafieyan et al. [129]	A total of 28 ML algorithms were applied to determine the most suitable one.	Prediction of cell differentiation and behavior in different cardiac tissue-engineered scaffolds.	A cardiac tissue-engineered scaffold dataset was created, and then the cardiac tissue-engineered scaffolds were rated based on cell behavior. Finally, 28 ML algorithms were utilized to conclude which one was the most successful in predicting cell behavior on cardiac tissue-engineered scaffolds.	The highest-performing individual algorithm was XGBoost, achieving an accuracy of 87%. The highest-performing algorithms for ensemble learning were Adaboost Classifier and VotingClassifier, achieving an accuracy of 93%. The results show that ML can accurately predict cell behavior on cardiac tissue-engineered scaffolds.
Conev et al. [130]	ML-based software (Random Forests) that utilizes a direct classification-based approach and an indirect approach.	Prediction of printing quality given the printed conditions in 3D bioprinting of scaffolds used for bone tissue engineering. Proposal of the most suitable parameters affecting the quality of the print for dataset construction.	The ML software used polymeric biomaterials and different printing configurations as inputs to determine if the resulting product quality was high or low. They used two different metrics to assess the printing quality, material accuracy, and machine precision.	Both models were able to correctly label the majority of the tested configurations. The most important parameters affecting the quality of a print were material composition, printing speed, and printing pressure. The results indicate the potential of ML for identifying suitable printing parameters and reducing experimentation.
Barrera et al. [131]	3D convolutional neural networks.	Prediction of tissue-engineered scaffolds’ mechanical properties with a more efficient and time-saving method by utilizing trained 3D convolutional neural networks.	The software is trained by inputting 2D-layered images of digital tomographies from the computer-aided design models and values from computer-aided design measurements and Finite Element Method simulations. They were utilized to predict the properties of a new set of scaffolds. Their performance has been assessed by the already trained 3D convolutional neural networks.	This methodology is a promising future aspect as a cost-effective tool for predicting the mechanical properties of 3D scaffolds and has the capability of being constantly updated by an expanding dataset.
Martin et al. [126]	NKLR (Norwegian Knee Ligament Register) ML-based tool.	External validation of the NKLR ML predictive models for ACL revision risk by evaluating their performance on different groups of patients from the Danish Knee Ligament Registry (DKLR).	10,922 DKLR patients were used, which satisfied five variables. The predicted revision probabilities were calculated for all DKLR patients for ACL revision. Finally, the tool’s performance was evaluated using the same metrics as the NKLR study.	The tool produced similar results when applied to the DKLR population compared to the original NKLR database (DKLR: 0.68; NKLR: 0.68–0.69). This suggests that the algorithm can be applied outside of the initial patient population and represents the first predictive ML model that has been externally validated for ACL reconstruction revision.
Ye et al. [128]	Different ML-based software.	Identifying the best-performing machine learning models for predicting the objective and subjective clinical outcomes of ACL reconstruction and determining the most important predictors.	A total of 432 patients who underwent ACL reconstruction were included. A variety of ML models were used in order to conclude which software had the highest performance for each clinical outcome (graft failure, residual laxity, PROMs, and return to sports).	The results were determined based on the area under the curve and the accuracy. The most successful results were as follows: (1) Graft failure: 0.944 (excellent) and 98.6%. (2) Residual laxity: 0.920 (excellent) and 91.4%. (3) Failure to achieve the minimal clinically important difference of the Lysholm score: 0.930 (excellent) and 91.0%. (4) Failure to achieve the minimal clinically important difference of the International Knee Documentation Committee score: 0.942 (excellent) and 95.1%. The results provide reliable predictions for the outcome of the ACL reconstruction surgery.
